# Differentiating Melancholic and Non-melancholic Major Depressive Disorder Using Fractional Amplitude of Low-Frequency Fluctuations

**DOI:** 10.3389/fpsyt.2021.763770

**Published:** 2022-02-02

**Authors:** Yingying Zhang, Xilong Cui, Yangpan Ou, Feng Liu, Huabing Li, Jindong Chen, Jingping Zhao, Guangrong Xie, Wenbin Guo

**Affiliations:** ^1^National Clinical Research Center for Mental Disorders, Department of Psychiatry, The Second Xiangya Hospital of Central South University, Changsha, China; ^2^Department of Radiology, Tianjin Medical University General Hospital, Tianjin, China; ^3^Department of Radiology, The Second Xiangya Hospital of Central South University, Changsha, China; ^4^Department of Psychiatry, The Third People's Hospital of Foshan, Foshan, China

**Keywords:** melancholic depression, non-melancholic depression, major depression disorder, fractional amplitude of low-frequency fluctuations, resting-state fMRI

## Abstract

**Background:**

Melancholic major depressive disorder (MDD) is a network-based brain disorder. However, whether or not network-based changes can be applied to differentiate melancholic (MEL) from non-melancholic (NMEL) MDD remains unclear.

**Methods:**

Thirty-one MEL patients, 28 NMEL patients, and 32 matched healthy controls (HCs) were scanned using resting-state functional magnetic resonance imaging. Patients were assessed by the Chinese version of Snaith–Hamilton Pleasure Scale (SHAPS-C) and Temporal Experience of Pleasure Scale (TEPS). Fractional amplitude of low-frequency fluctuations (fALFF) and correlation analysis were used to analyze the data.

**Results:**

Compared with HCs, the MEL group had significantly higher fALFF values in the bilateral inferior frontal gyrus and right supplementary motor area (SMA) and significantly lower fALFF values in the right inferior occipital gyrus (IOG), right middle temporal gyrus (MTG)/left IOG, and bilateral superior occipital gyrus (SOG)/MTG. On the other hand, the NMEL group showed significantly higher fALFF values in the bilateral SMA and significantly lower fALFF values in the bilateral posterior cingulate cortex/precuneus relative to HCs. Compared with the NMEL group, the MEL group showed significantly lower fALFF values in the left anterior cingulate cortex (ACC). A correlation was found between the fALFF values of the right SMA and the SHAPS-C in the MEL group. In addition, correlations were observed between the fALFF values of the left ACC and the TEPS contextual consummatory and total scores in all patients.

**Conclusion:**

Our study uncovered that MDD exhibited altered brain activity in extensive brain networks, including the default-mode network, frontal-striatal network, reward system, and frontal-limbic network. Decreased fALFF in the left ACC might be applied to differentiate the two subtypes of MDD.

## Introduction

Major depressive disorder (MDD), a common mental disorder, is marked by a persistent negative mood, anhedonia (1), cognitive impairments (2), and additional symptoms, such as psychomotor changes, appetite dropping, sleep disturbance, fatigue, inattention, and sense of valuelessness (3). As the most important precursor of suicide (4), MDD was ranked as the first global disease burden in middle- and high-income countries in 2008 (5). On the basis of DSM-IV criteria and recent studies, melancholic (MEL) MDD is a severe subtype of MDD, also known as primary, typical, or endogenous depression (3, 6–10). Distinct from non-melancholic (NMEL) MDD, MEL MDD is characterized by MEL features of anhedonia (refers to impaired mood reactivity, reward dysfunction, and diminished interest), psychomotor disturbance, and cognitive impairment (including attention shift, visual learning, implicit learning, and executive functions) (3, 6, 9–19). Psychomotor retardation is the chief characteristic of MEL MDD (10, 13, 20). However, the pathophysiology underlying MEL MDD remains unclear.

Different brain regions are responsible for certain specific functions and typical clinical symptoms, and some studies gradually focus on brain regions related to MEL features. Psychomotor retardation is an important MEL feature, and it involves the primary motor, premotor and supplementary motor area (SMA), posterior parietal lobe (21), and cerebellum (22). Recent resting-state fMRI studies have reported that the SMA functions in cognitive and emotional domains (23–26) and has abnormal spontaneous activity in patients with MDD (27). Reductive SMA volume contributes to implicit learning deficits in MEL patients (20). Anhedonia, a core MEL feature, is related to the anterior cingulate cortex (ACC), ventral striatum (VS), and frontal gyrus. Recent studies have found that the hypoactivation of the VS could reflect anhedonia severity (19, 28, 29), and functional connectivity (FC) of the subgenual ACC is significantly associated with depressive episode duration (30, 31). Cognitive function, another important MEL feature (16), is related to the frontal gyrus, superior temporal gyrus (STG) (16), posterior cingulate cortex (PCC) (32), middle occipital gyrus (MOG), and other brain regions (33).

MDD is a complex network-based disorder rather than a dysfunction in specific brain regions (29). The default mode network (DMN) (18), frontal-limbic network (34, 35), reward system (17, 36, 37), and frontal-striatal network (6, 38) are related to MEL features. Previous studies indicated that MEL patients express a decreased spontaneous activity in the DMN (6) and have a correlation between alterations of the DMN and anhedonia severity (18). Aside from being related to the SMA, implicit learning is supported by the frontal-striatal network (39, 40), and functional abnormalities of the frontal-striatal network have been observed in patients with MDD (41). Although this network is closely linked to MEL MDD (20, 42, 43), few studies exactly verified the hypothesis (20). As for the other networks, multiple studies focused on MDD, but few focused on MEL MDD. The frontal-limbic network was related to symptomatic mood disorder (44), vegetative features, and cognitive dysfunction (35), and recent studies have observed opposite trends of limbic and frontal areas in patients with MDD (29, 35, 45). Current MRI studies also found alterations of reward-related areas in MDD (41, 46), and reward dysfunction might contribute to the mood reactivity deficits and other MEL features (17, 19). However, a consistent result has yet to be reached.

In recent decades, neuroimaging has gradually become a crucial way to explore the etiology and pathogenesis of mental diseases (47). Some studies have focused on the pathogenesis of MDD (48). Given the prevalence and persistence of depressive symptoms, multiple functional magnetic resonance imaging (fMRI) has been developed to explore brain alterations in resting state (49, 50). Blood oxygenation level-dependent (BOLD) fMRI has been used to estimate oxygen consumption and blood inflow (47). Amplitude of low-frequency fluctuation (ALFF) and fractional ALFF (fALFF) use BOLD-based signals to identify the low-frequency oscillation (LFO) amplitude in resting state (42, 51), reflecting the absolute intensity of spontaneous intrinsic brain activity to the area. With the development of fALFF, non-specific area artifacts can be suppressed (52), making it effective in normal and pathological brains (53, 54).

Several studies used the ALFF/fALFF method to analyze the brain spontaneous activity of MDD, but none were able to distinguish MEL patients from NMEL patients. Altered fALFF values have been discovered in extensive brain regions of patients with MDD, such as the left frontal gyrus (22, 32, 50, 55, 56), right middle frontal gyrus (22, 32, 35, 56, 57), right superior temporal gyrus (22), left middle and inferior temporal gyrus (22, 49, 50, 55, 58), right superior parietal postcentral lobe (55, 56), right middle occipital gyrus (49, 56), fusiform gyrus (49, 59), right striatum (22), limbic system [including ACC (21) and PCC/PCu] (57, 60, 61), bilateral parahippocampal gyrus (42, 50, 59, 61), left thalamus (32), and bilateral cerebellum lobe (22, 49, 59). However, the results are sometimes inconsistent because of various reasons, such as a small sample size and heterogeneity of subjects (21). Furthermore, studies have found a correlation between altered fALFF values in certain brain regions (e.g., the right rostral ACC, right PCu, left thalamus, and left somatosensory cortex) and depressive severity (32, 42), suggesting the possibility of using fALFF as an indicator.

Combining all the above mentioned studies, the present study aimed to (1) utilize the fALFF method to obtain the different values across three groups [MEL, NMEL, and matched healthy controls (HCs)] and compare brain function between groups and to (2) investigate the correlations between significantly abnormal fALFF values and anhedonia severity as indicated by two neuropsychological scales, the Temporal Experience of Pleasure Scale (TEPS) and Snaith–Hamilton Pleasure Scale (SHAPS) to identify whether or not a correlation exists between altered brain functions and clinical symptoms. We hypothesized that (1) all patients exhibited altered brain fALFF values in extensive brain networks and that (2) an association might exist between altered fALFF values and anhedonia severity, reflected by the scores of TEPS and SHAPS.

## Materials and Methods

### Participants

A total of 31 MEL outpatients and 33 NMEL outpatients with age ranging from 18 to 45 participated in this study. The patients were all recruited from the Second Xiangya Hospital, Central South University, Changsha, China. This study lasted from May 4, 2014 to December 30, 2016. The diagnosis was authoritatively confirmed by two psychiatrists in accordance with the DSM-IV criteria. Thirty-two HCs were recruited from the general public through advertisement.

All patients met the following inclusion criteria: (1) first episode of MDD with the score of Hamilton Rating Scale for Depression (HRSD-17) more than 17; (2) illness duration no more than 12 months; and (3) having no history of psychotic medication or electroconvulsive therapy. The diagnostic criteria of MEL MDD in the DSM-IV criteria were required as follows: (1) more than or equal to one of the following symptoms occurs in the most severe period of the current episode: loss of pleasure in almost all activities (also known as pervasive anhedonia) and lack of mood reactivity to usually pleasurable stimuli (does not feel a little better even something good happens or non-reactive mood); (2) at least three of the following symptoms: distinct quality of depressed mood (i.e., depressive mood experienced is qualitatively different from the feeling experienced when the loved one dies); showing extreme despondency, despair, and/or morose mood or alleged empty mood); often more severe in the morning; early morning awakening (i.e., at least 2 h earlier than usual awakening) (HRSD item 6 ≥ 1); characterized psychomotor agitation or marked retardation (HRSD items 8 or 9 ≥ 2); salient weight loss or anorexia (HRSD items 12 or 16 = 2); and excessive or improper guilt (HRSD item 2 ≥ 2). Patients who fell short of these criteria were assigned to the NMEL group.

Age and sex of patients were matched with those of HCs. Potential HCs with any neurological diseases, psychiatric symptoms, or substance abuse were excluded. Potential HCs whose first-degree relatives had a history of mental illness were also excluded. Exclusion criteria were as follows: (1) other mental disorders involved in the DSM-IV criteria; (2) ever suffered from neurological disorders, grievous somatic illnesses, or substance abuse; (3) pregnancy; (4) abnormal brain structure as original MRI scan finding; and (5) contraindications for MRI scan.

All participants were right-handed and Han Chinese with at least 9 years of education. The severity of depression was determined by the HRSD-17; the anxiety state was evaluated using the Beck anxiety inventory (BAI); the anhedonia state was assessed by using the SHAPS-C—the higher the score, the more severe the anhedonia (62)—for all patients. The Chinese version of TEPS was applied to capture the level of anticipatory and consummatory facets of pleasure in all patients—the lower the score, the greater the anhedonia.

This study passed the assessment of the Medical Research Ethics Committee of the Second Xiangya Hospital, Central South University, Changsha, China. The study was conducted in accordance with the Helsinki Declaration. All participants signed a written informed consent.

### Image Acquisition

Resting-state MRI data were acquired with a 3.0 T Siemens scanner (Germany). Resting-state fMRI images were acquired by a spoiled gradient recall sequence, and the parameters of the echo planar imaging (EPI) sequence were set as follows: repetition time/echo time (TR/TE) as 2,500/25 ms; 39 slices; matrix as 64 × 64; flip angle as 90°; field of view as 240 × 240 mm; slice thickness as 3.5 mm; no gap; and volumes as 200. The scan lasted for 500 s. All participants were instructed to remain still, supine, remain their eyes closed, and stay awake. During scanning, soft earplugs and quadrature birdcage coil with foam padding were used to minimize noise and head movement.

### Data Preprocessing

The DPABI software was used to conduct the data preprocessing and statistical analysis of functional images (63). In view of the influence of the initial MRI signal's instability and patients' adaptation period, the first 10 images were discarded to minimize the impact, and the remaining 190 volumes were corrected of slice acquisition delays and subject head motion. Participants were included only when any displacement in the x, y, or z axis was <2 mm and angular rotation was <2° (61). The corrected images were normalized to the standard Montreal Neurological Institute (MNI) space as 3 × 3 × 3 mm. Then, spatial smoothing was executed to the normalized images with an 8-mm full-width at half maximum Gaussian kernel (61). Finally, linear trend and temporal filtering (0.01–0.08 Hz) were conducted on the time series of each voxel to reduce the effect of low-frequency drifts and physiological high-frequency respiratory and cardiac noise for further fALFF analysis.

### fALFF

In accordance with a previous study (52), the fALFF analysis using an in-house software REST (http://www.resting-fmri.sourceforge.net) was performed as follows. First, fast Fourier transform was used to convert the time course of each voxel to the frequency domain without band-pass filter, obtaining the power spectrum. Second, the square root at each frequency of the power spectrum was calculated, given that a certain frequency power was proportional to the square of its amplitude, and the averaged square root was obtained across 0.01–0.08 Hz at each voxel. Third, for the purpose of standardization, the sum of amplitudes across 0.01–0.08 Hz was divided by that across the complete frequency range. On the basis of a brain mask (49), the fALFF in each voxel would be divided by the global mean fALFF value. The average fALFF values were extracted using REST.

### Neuropsychological Scales: TEPS and SHAPS-C

The TEPS is a measure assessing the sense of anhedonia and evaluating one's long-term experience of pleasure simultaneously. The original English version contains 18-item, six-point Likert format, having good internal consistency and test–retest reliability. Considering the cultural differences and referring to two doctorate degree experts' opinions, the current study used a 20-item Chinese version (62, 64). Compared with the original English version, two items were excluded (Item 5 “I love it when people play with my hair” and Item 11 “When I'm on my way to an amusement park, I can hardly wait to ride the roller coasters”), and two items (one anticipatory item and one consummatory item) were added in the final Chinese version of TEPS (64). Thus, far, many studies have been carried out to prove the reliability of the Chinese version of TEPS (62, 64, 65).

SHAPS (66) is a 14-item checklist used to assess the anhedonia and positive valence for the current study (1). This scale measures the patients' state during the last 2 weeks. It was a four-point Likert scale, which ranges from absolute agree to absolute disagree rather than the original dichotomy (agree and disagree scores 0 and 1) proposed by Snaith et al. (66). In the Chinese version of SHAPS (SHAPS-C), the total score ranges from 14 and 56. This method helps improve the dispersion of the data for internal consistency, structure validity, and strengthen the convergent correlations between SHAPS and other scales (62).

### Statistical Analysis

ANOVA was performed using SPSS20.0 to access the differences in age and education years across the three groups. Two-sample *t*-tests were performed to compare group differences in the course of disease, SHAPS-C scores, and TEPS scores between the MEL and NMEL groups. A Chi-square test was used to access the gender distribution.

Micro-movement based on the framewise displacement (FD) measurement was calculated (67). Analysis of covariance (ANCOVA), followed by *post-hoc t*-tests, was conducted to compare fALFF differences across groups. Age, gender, education years, and FD values were used as covariates for reducing the possible impact of these factors. Gaussian random field theory was utilized for correction by setting the significance threshold to *P* < 0.05 (voxel significance as *P* < 0.001 and clustering significance as *P* < 0.05).

Linear correlation analyses were performed to identify whether the fALFF values in certain regions that were significantly different between groups related to the anhedonia-related neuropsychological assessment, TEPS, and SHAPS. Bonferroni correction was applied to correct multiple correction and improve the inspection level.

## Results

### Participants

Data from five NMEL patients were excluded because of excessive head movement. As indicated in Table 1, a total of 31 MEL patients, 28 NMEL patients, and 32 HCs finally completed the whole study. The three groups did not significantly differ in age (*p* = 0.107), gender (*p* = 0.461), and handedness, and no significant differences in illness duration (*p* = 0.500) were found between the two patient groups. The three groups expressed significant differences in terms of the year of education, HRSD-17, BAI, and SHAPS-C scores. The education level (*p* = 0.003) of the NMEL group was significantly lower than that of the MEL and HC groups. The HRSD-17 (*p* < 0.001), BAI (*p* < 0.001), and SHAPS-C scores (*p* < 0.001) of the MEL and NMEL groups were significantly higher than those of the HC group. Significant differences in TEPS total scores, TEPS abstract anticipatory scores, and TEPS contextual anticipatory scores were observed between the patient groups. The TEPS total scores (*p* = 0.002), TEPS abstract anticipatory scores (*p* = 0.001), and TEPS contextual anticipatory scores (*p* = 0.001) were significantly higher in the NMEL group than in the MEL group. Meanwhile, no significant differences in TEPS abstract consummatory scores (*p* = 0.117) and TEPS contextual consummatory scores (*p* = 0.074) were observed between two patient groups. Additional details are exhibited in Table 1.

**Table 1:** Demographic and clinical characteristics of the participants.

	**Melancholic (*n* = 31)**	**Non-melancholic (*n* = 28)**	**Healthy controls (*n* = 32)**	***F*, *t* or *χ^2^* value**	***P*-value (two-tailed)**
Age (years)	28.65 ± 5.30	32.04 ± 8.18	29.59 ± 5.00	2.291	0.107^a^
Gender (male/female)	10/21	10/18	15/17	1.55	0.461^b^
Handedness (right/left)	31/0	28/0	32/0		
Education (years)	15.16 ± 3.20	12.54 ± 3.00	14.59 ± 2.82	6.143	0.003^a^
Illness duration (months)	6.75 ± 4.26	5.96 ± 4.64		−0.68	0.500^c^
HRSD-17 scores	21.77 ± 3.79	21.00 ± 3.14	0.94 ± 0.95	527.891	<0.001^a^
BAI scores	44.00 ±11.51	38.77 ± 9.84	22.63 ± 2.28	50.895	<0.001^a^
SHAPS-C scores	37.23 ± 6.04	31.89 ± 5.24	21.59 ± 5.36	64.191	<0.001^a^
TEPS total scores	58.30 ± 14.19	69.46 ± 11.16		−3.315	0.002^c^
TEPS abstract anticipatory	13.17 ± 4.79	17.04 ± 3.85		−3.373	0.001^c^
TEPS contextual anticipatory	13.13 ± 3.96	16.68 ± 3.64		−3.540	0.001^c^
TEPS abstract consummatory	20.20 ± 5.21	22.39 ± 5.28		−1.592	0.117^c^
TEPS contextual consummatory	11.80 ± 3.23	13.36 ± 3.27		−1.824	0.074^c^

*HRSD-17, 17-item Hamilton Rating Scale for Depression; BAI, Beck anxiety inventory; SHAPS-C, Chinese version of Snaith–Hamilton Pleasure Scale*.

a *p-values were obtained by analyses of variance*.

b *p-value was obtained by a chi-square test*.

c *p-values were obtained by two-sample t-tests*.

### Differences in fALFF Values of Global Brain Regions Across Patients With Melancholic MDD, Non-melancholic MDD, and Healthy Controls

**Figure 1:**
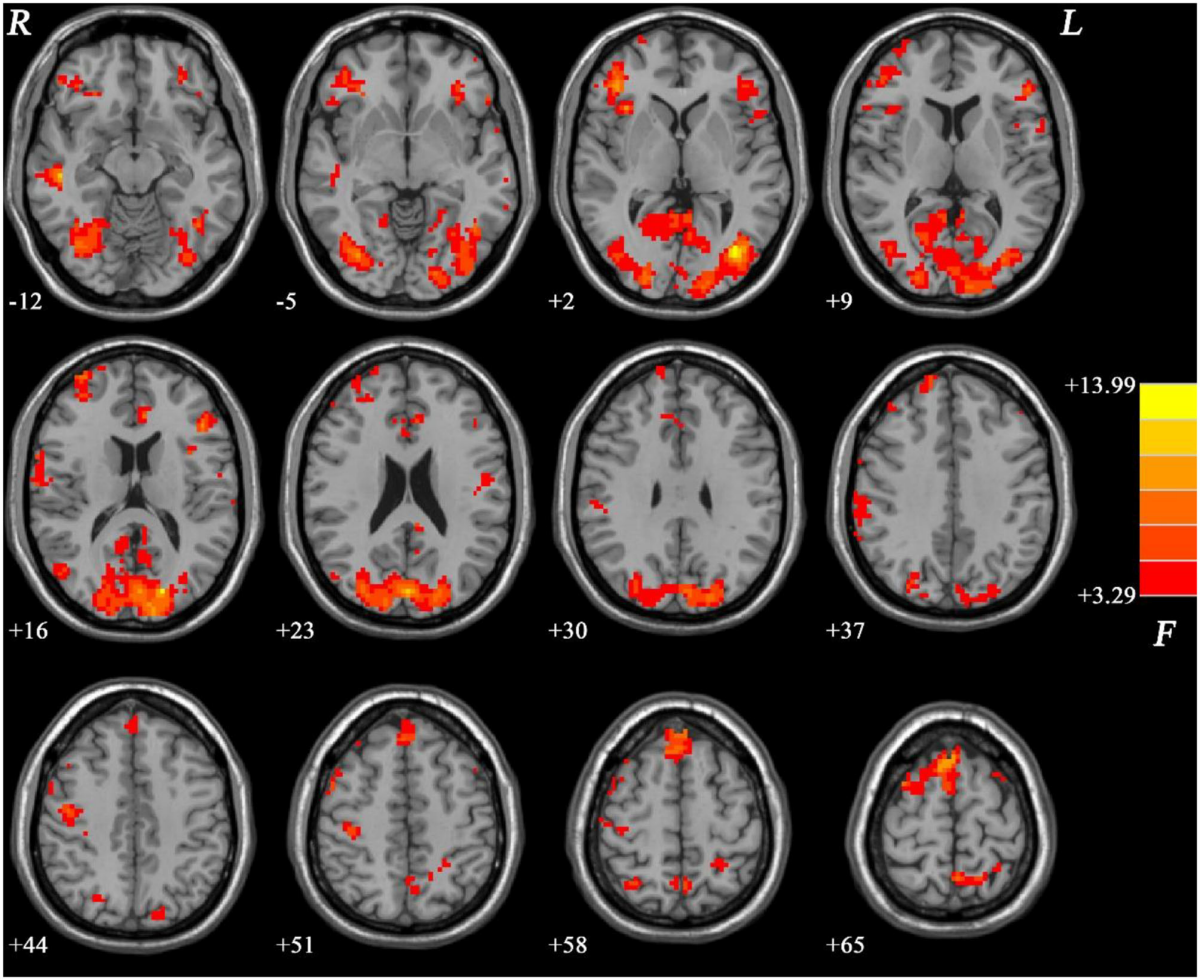
ANCOVA results (age, years of education, and framewise displacement as covariates) showing group differences of fALFF values across three groups in brain regions. Color bar indicates *F*-values of ANCOVA. fALFF, fractional amplitude of low-frequency fluctuations; ANCOVA, analysis of covariance.

**Figure 2:**
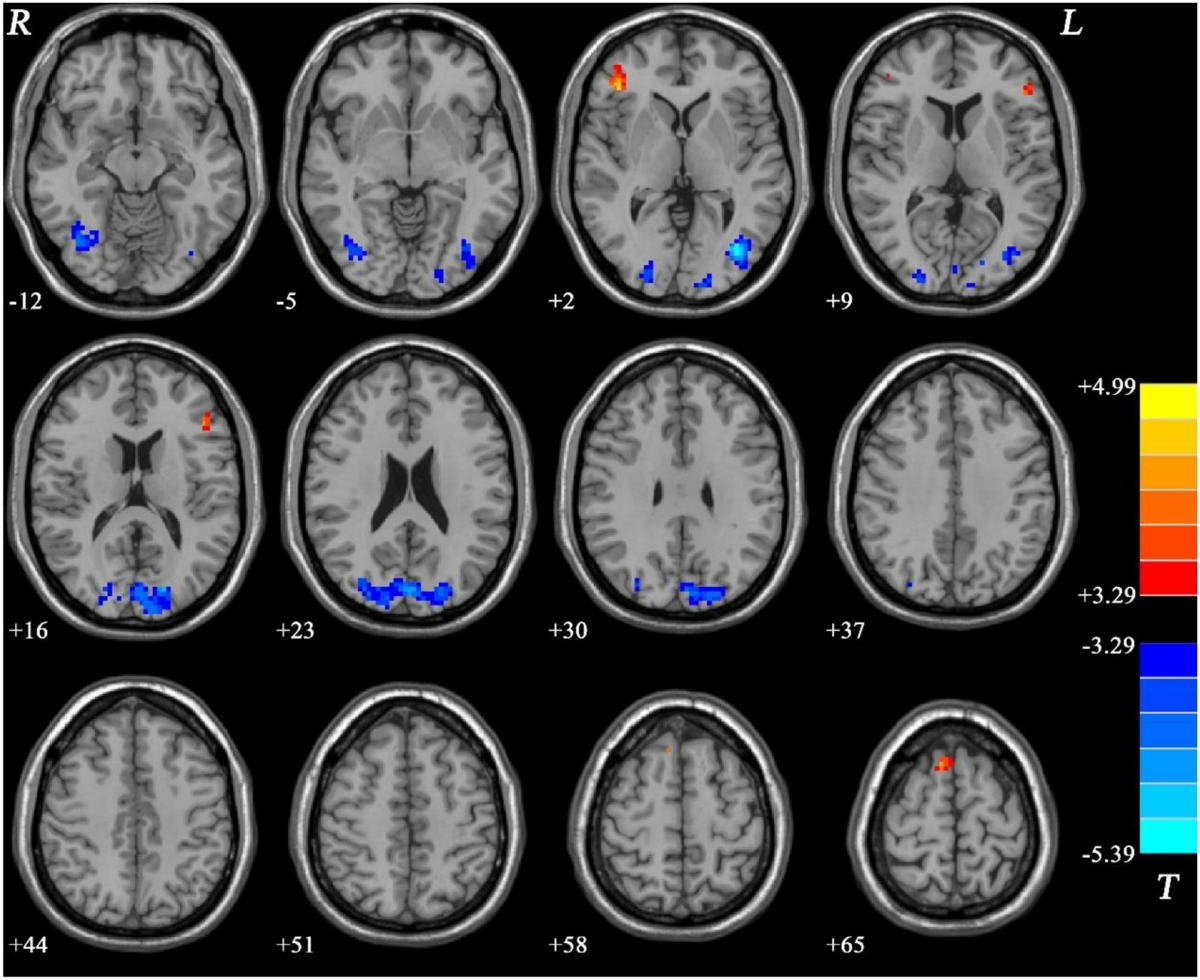
Fractional amplitude of low-frequency fluctuation (fALFF) differences between melancholic major depressive disorder (MDD) and healthy controls (HCs). Red and blue represent increased and decreased regional spontaneous activity in the patients, respectively.

**Figure 3:**
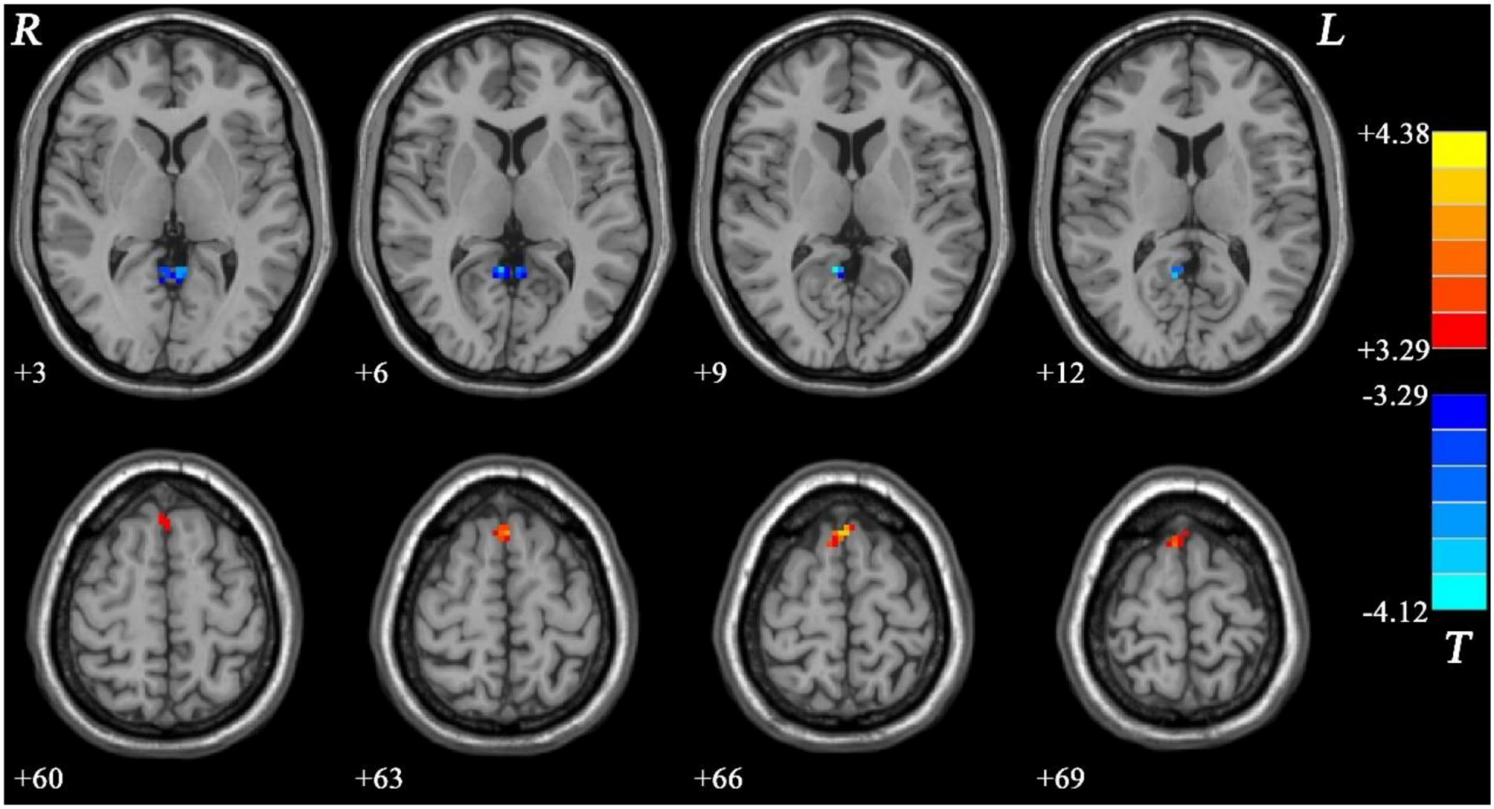
Fractional amplitude of low-frequency fluctuation (fALFF) differences between non-melancholic major depressive disorder (MDD) and healthy controls (HCs). Red and blue represent increased and decreased regional spontaneous activity in the patients, respectively.

**Figure 4:**
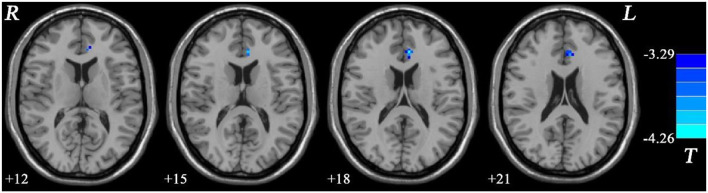
Fractional amplitude of low-frequency fluctuation (fALFF) differences between melancholic major depressive disorder (MDD) and non-melancholic major depressive disorder (MDD). Blue represents decreased regional spontaneous activity in the melancholic patients.

Group differences are shown in Table 2, Figures 1–4. Analysis of ANCOVA exhibited significant differences of fALFF mainly in the bilateral inferior frontal gyrus (IFG), bilateral SMA, right middle temporal gyrus (MTG)/left inferior occipital gyrus (IOG), right IOG, bilateral superior occipital gyrus (SOG)/MTG, left ACC, and bilateral PCC/precuneus (see Figure 1).

**Table 2:** Significant fALFF differences across groups.

**Cluster location**	**Peak (MNI)**			**Number of voxels**	***T*-value**
	**x**	**y**	**z**		
**Melancholic vs. Healthy controls**					
Right inferior occipital gyrus	36	−69	−12	74	−4.3247
Left middle temporal gyrus/inferior occipital gyrus	−36	−75	3	116	−5.3940
Bilateral superior occipital gyrus/middle temporal gyrus	−18	−84	15	482	−4.9707
Right inferior frontal gyrus	42	36	0	43	4.9948
Left inferior frontal gyrus	−45	27	15	24	4.2466
Right SMA	9	18	69	32	4.6609
**Non-melancholic vs. Healthy controls**					
Bilateral PCC/precuneus	9	−51	9	27	−4.1203
Bilateral SMA	0	21	66	34	4.1208
**Melancholic vs. Non-melancholic**					
Left anterior cingulate cortex	−6	33	18	20	−4.2649

*fALFF, fractional amplitude of low-frequency fluctuations; MNI, Montreal Neurological Institute; PCC, posterior cingulate cortex; SMA, supplementary motor area; the p-values are the peak voxel significance*.

Compared with the HC group, the MEL group exhibited significantly lower fALFF values in the right IOG, right MTG/left IOG, and bilateral SOG/MTG and significantly higher fALFF values in the bilateral IFG and right SMA (see Figure 2, Table 2).

Meanwhile, the NMEL group showed significantly lower fALFF values in the bilateral PCC/precuneus and significantly higher fALFF values in the bilateral SMA relative to the HC group (see Figure 3, Table 2).

In addition, the MEL group showed significantly lower fALFF values in the left ACC than the NMEL group (see Figure 4, Table 2).

### Correlations Between fALFF and the Scales Scores (TEPS and SHAPS-C)

**Figure 5:**
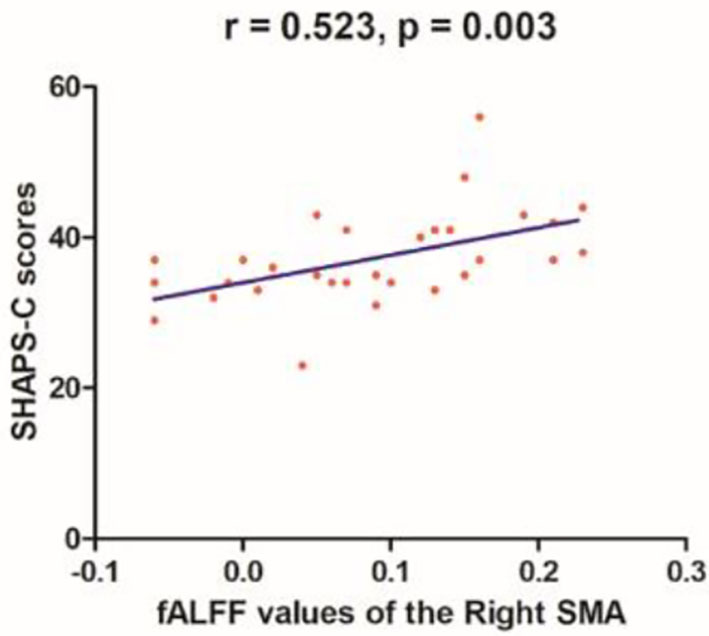
A correlation between fALFF values of the right supplementary motor area (SMA) and SHAPS-C scores (*r* = 0.523, *p* = 0.003) in the melancholic patients.

**Figure 6:**
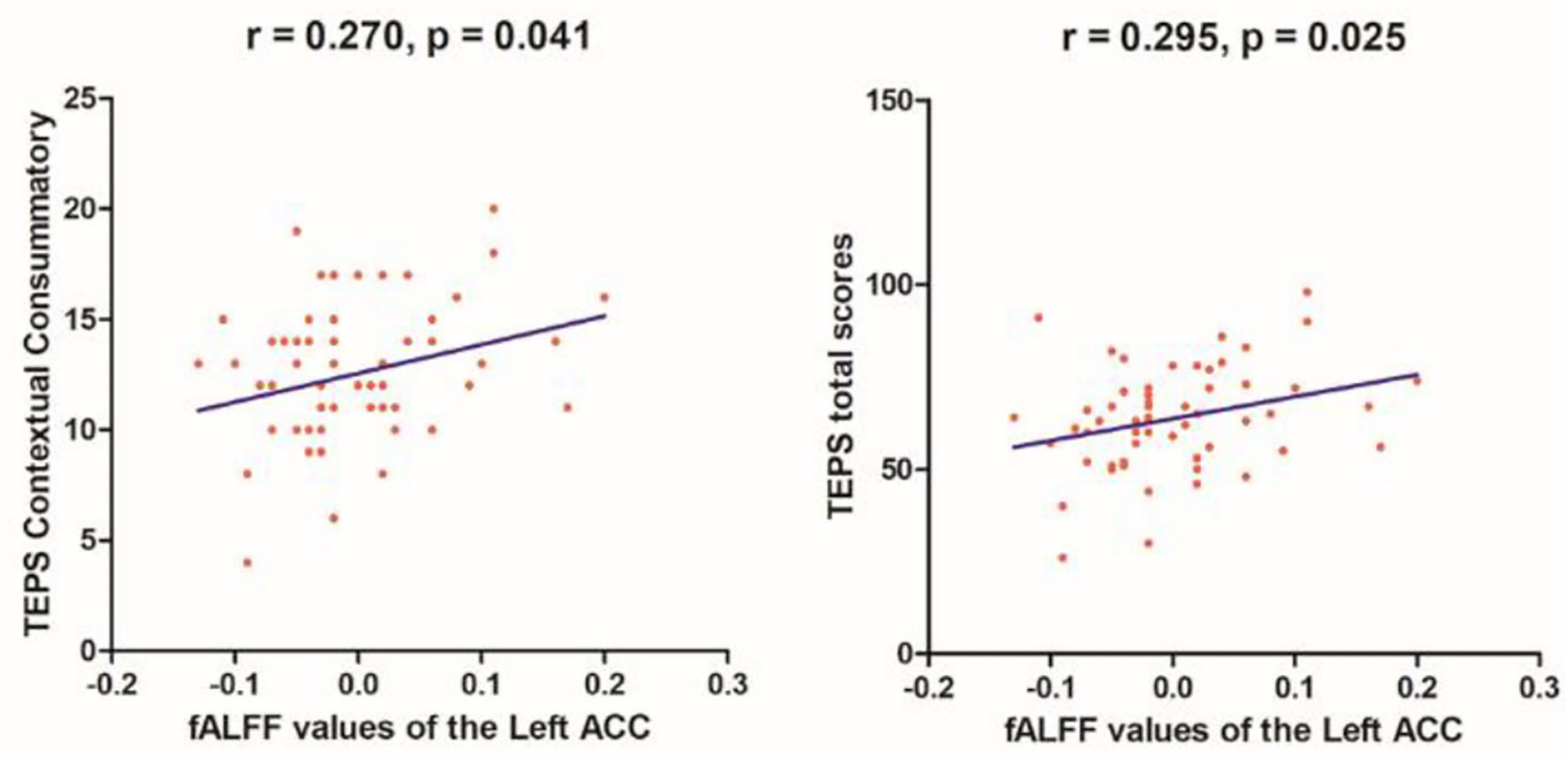
Correlations between fALFF values of the left anterior cingulate cortex (ACC) and TEPS contextual consummatory scores (*r* = 0.270, *p* = 0.041) and TEPS total scores (*r* = 0.295, *p* = 0.025) in all patients.

Linear correlation analyses indicated significant association between fALFF values in the right SMA (*r* = 0.523, *p* = 0.003) and SHAPS-C scores (see Figure 5) in the MEL group but not in the NMEL and HC groups. In all patients, linear correlation analyses indicated significant associations between fALFF values in the left ACC and TEPS contextual consummatory scores (*r* = 0.270, *p* = 0.041) and TEPS total scores (*r* = 0.295, *p* = 0.025) (see Figure 6).

## Discussion

In the present study, all patients exhibited altered brain activity in extensive brain networks, including the default-mode network, frontal-striatal network, reward system, and frontal-limbic network. A correlation was found between the fALFF values of the right SMA and the SHAPS-C in the MEL group. In addition, correlations were observed between the fALFF values of the left ACC and the TEPS contextual consummatory and total scores in all patients.

Network models express the interactions among brain regions (68), which are crucial to learn the pathophysiology of complex disease phenotypes (12, 69, 70). MDD is a network-based disorder (70–73), and high-traffic network hubs are vulnerable to pathological hyperstimulation (18, 74, 75), leading to a decline in brain function in related areas (33). MEL MDD is regarded as a severe subtype of MDD, tending to display characteristics as anhedonia (including reduced emotional reactivity, reward dysfunction, and decreased interest), psychomotor retardation, and greater cognitive deficits (especially speed of processing and executive functions), and psychomotor retardation has been regarded as a major feature (3, 6, 9–20, 76, 77). Four networks were proposed closely related to MEL features (refer to the DMN, frontal-limbic network, reward network, and frontal-striatal network).

Several brain regions, such as the frontal gyrus and ACC play considerable roles in MEL features. The frontal gyrus, as a hub of executive control functions, plays a crucial role in all these networks and influences the visual memory and problem solving in MEL MDD (16). As a critical part of the motor area in the frontal gyrus (78), the SMA is characterized by unique motor function [refers to self-initiated (20) and helplessness-related motor behavior (79)] and special non-motor functions of cognitive control (majoring in the working memory) (23), sensorimotor integration (78), and task-switching (80, 81). Given that psychomotor retardation is a distinct characteristic in MEL MDD, alterations in motor areas, especially the SMA, increasingly became the focus of recent studies. A number of studies reported the importance of the SMA in motor learning (78), emphasizing the correlation between SMA and implicit learning in MEL patients. Implicit learning deficits are closely related to altered SMA, and recent studies have suggested that implicit learning is mainly supported by the frontal-striatal network, including the SMA, premotor area, and ACC, additionally majored in learning skill, purposive behavior, and adaptability (39, 40). This network is highly closely linked to MEL MDD (20, 42, 43), but no verified conclusion has been reached (20). Schmaal et al. (82) observed delayed mature SMA in adolescent patients. Yang et al. (83) found altered fALFF values in the left motor cortex. A consistent result was obtained that both MEL and NMEL patients express reduction in the SMA, whereas the right SMA area of MEL patients is most pronounced (20, 34, 81). The right motor cortex was suggested connected with global brain regions (81), such as the middle frontal lobe, the STG, and the basal ganglia (78). In our study, both MEL and NMEL groups showed increased fALFF in the right SMA, whereas the fALFF in the left SMA only significantly increased in the NMEL group. Thus, SMA could play a special role in patients with MDD, and altered fALFF in this area might correlate to the function deficits in patients with MDD, such as autonomous behavior, implicit learning, cognition, and adaptability to changed conditions. Our findings provided a possibility that the significantly changed fALFF in the right SMA might be a breakthrough point to investigate the characteristics of MEL patients.

The orbitofrontal cortex (OFC), containing in the IFG, is a crucial region for emotional and cognitive control. The OFC is involved in the reward system, linking reward to happy experience (36), which contains the VS, ACC, ventral tegmental area (VTA), nucleus accumbens, and dorsolateral prefrontal cortex (36, 37), contributing to impaired mood reactivity and MEL features (17, 19). Schneider et al. (84) found size reduction in the left IFG; Guo et al. (35) observed decreased brain activity in the OFC of patients with MDD; and Bracht et al. (36) detected decreased connectivity between the right VTA and the OFC in MEL patients. These results are unexpectedly different from our findings that regional activity increased in the bilateral IFG of MEL patients. The discrepancies of results might be due to the small sample size, subject heterogeneity, and different research methods in these studies (21), which suggested the importance of homogenous subgroups in further investigations. Furthermore, the right IFG has emphasized the importance in function of transmitting mood/behavior information and connecting with brain regions. Roberts et al. (85), Rolls et al. (86), and Heather et al. (87) revealed its associations to other frontal regions, including the insula, putamen, precuneus, and supracallosal ACC (known as punishment area). Cui et al. found its decreased effective connectivity to the insula in MEL patients (6). Consistent with our result, altered fALFF of the IFG in MEL patients might suggest abnormal cognitive and emotional functions in the patients.

Anhedonia is a core symptom of melancholia, contributed by various dysfunctions in networks, especially the frontal-striatal network (6, 38), reward network (36, 37), DMN, and emotional behavior modulation circuits (18). Brain regions in the reward system are mostly included in the frontal-striatal network (36, 37), and striatum and ACC are shared by the frontal-striatal network, reward network, and ventral emotional circuitry (18, 19, 88). Current studies revealed a hypoactivation that the VS could reflect anhedonia severity (19), and the ventral emotional circuitry shows an elevated connectivity in NMEL patients, whereas a reduced connectivity is observed in MEL patients (18).

The DMN, which contains the MPFC, ACC, PCC/PCu, parietal cortex, lateral temporal cortex, and angular gyrus (6, 18, 42, 89), mainly functions on emotional processing and self-referential activities (90–93), with reduced reactivity to stimuli, also indicating anhedonia severity (18) and aberration in numerous MDD studies (6, 42, 94). In our study, we found lower fALFF values in the left ACC of the MEL group compared with the NMEL group and in the bilateral PCC/precuneus of the NMEL group compared with the HC group.

As mentioned above, previous studies have uncovered that the ACC is significantly related to anhedonia (95), suggesting the core role of ACC in MEL MDD (36, 96, 97). The ACC has additional functions, such as voluntary action (80), cognitive control, and mood regulation (95). Moreover, as a key part of the limbic system, the ACC plays a crucial role in the frontal-limbic network (containing the ACC, lateral and MPFC, OFC, VS, hippocampus, and anterior thalamus) (34, 35, 98) and is correlated with symptomatic mood disorder (44), vegetative and somatic features, and cognitive function (35). Guo et al. (35) observed hypofunction in the frontal gyrus and hyperfunction in the limbic system in patients with MDD. Many studies focused on ACC alterations in patients with MDD. Schmaal et al. (82) found cortical structural abnormality; Yao et al. (79) found decreased fractional anisotropy (FA); Tadayonnejad et al. (32) revealed that a relation exists between altered fALFF and depressive severity; and Zhao et al. (99) suggested that the synaptic transmission in ACC catalyzes suicide concept and behavior. Moreover, the ACC is connected to extensive brain regions, such as the bilateral MTG, VS, dorsal lateral PFC, and paralimbic regions (95), and Rolls (100) indicated that the connectivity from the OFC to the pregenual ACC could transmit reward/punishment information. Our result, when combined with previous results, suggests that abnormal fALFF in the ACC is tightly associated with anhedonia, which helps estimate the severity of anhedonia in patients with MDD. NMEL patients prefer to express anxiety, and the ACC is considered an anxiety-related region in patients with MDD (101). This report is in line with our result that the correlations between ACC and anhedonia severity show no significant differences between the MEL and NMEL groups.

As parts of DMN, the PCC/precuneus are effective in self-memory, prospection, situation retrieval, and psychological scene construction (102). In their studies on patients with MDD, Shi et al. (81) and Schneider et al. (84) found reduced functional networks; Abdallah et al. (103) found increased reveal global brain correlation; and Sun et al. (104) observed significantly abnormal regional homogeneity. Schmaal et al. (82) found that precuneus deficits are closely related to anxiety, and previous findings indicated that NMEL patients are more likely to suffer from anxiety (18, 105, 106). In line with abovementioned studies, our result that significantly decreased fALFF in the bilateral PCC/PCu in the NMEL group compared with the HC group supported the hypothesis that dysfunctions in PCC/PCu contribute to the anxiety clinical features in NMEL patients and help differentiate MEL from NMEL patients.

Furthermore, using the fALFF approach, we found decreased brain activity in the bilateral SOG, IOG, and MTG in patients with MEL compared with HCs. The SOG and the IOG are involved in the visual area, mainly controlling visual processes (107). Current studies have emphasized the functional roles of the visual system in patients with MDD. The SOG is a key region related to visual recognition (108). Chen et al. (109) and Garrett et al. (110) found a high autonomic activation of the SOG. Bonte et al. (111) found perfusion deficits in the SOG, and occipital asymmetry was uncovered by previous studies (107, 109). In addition, nerve pathways are decreased in the visual area of MDD (79), such as the left SOG to the dorsal attention network (109) and the right MOG to the amygdala (112), which are closely correlated with cognitive dysfunction. The abovementioned studies suggest that abnormal fALFF in the occipital gyrus is relevant to impaired visual learning, visual memory, and cognitive function in patients with MDD (16, 61).

The temporal gyrus is involved in the neural circuitry of emotion, cognition, and memory processing (6, 113, 114) and is correlated with suicide contempt (95). The temporal regions of patients with MDD show various structural and functional abnormalities, including gray matter volumetric reduction [related to depression severity (115)], lower regional activities, and altered FC (116–118). In MEL patients, current studies highlighted a rightward advantage of the temporal gyrus (119). Cui et al. (6) found decreased spontaneous activity in the right MTG/TP, and Glosser et al. (120) found an association between the right TP and emotion. Uniting these findings, altered temporal fALFF in our study indicated the depressive state of MDD, reflecting the severity of depression. Given that MEL is a severe state of MDD, this might possibly be a meaningful indicator of MEL, and the suicide risk of these patients is increased.

Moreover, when compared with the HCs, MEL patients expressed relation between altered fALFF values of the right SMA and the SHAPS-C scores, whereas both MDD groups showed associations between fALFF values of the left ACC and the TEPS contextual consummatory and total scores. Given that anhedonia was surmised a fundamental feature that helps differentiate MDD subtypes, as indicated by the SHAPS-C and TEPS scores in the present study, altered fALFF values in the ACC and SMA may be a potential marker to recognize MDD patients from HCs.

Inevitably, our study has the following limitations. First, the MEL and NMEL subtypes expressed different depressive degrees. Although under partial correlation calculation, identifying whether or not the alteration driven by MEL symptoms nor depressive severity is still confusing (10, 16, 36). Second, the sample size of each group is relatively small to classify NMEL patients into different subtypes. Third, recent studies have identified that respiratory and cardiac fluctuations and high ALFF in cisternal areas (52, 121) could cause physiological noises that influence the outcomes. Thus, we need to develop a strict approach to correct these noises in future studies.

## Conclusion

Our study showed that MDD exhibited altered brain activity in extensive brain networks, including the DMN, the frontal-striatal network, the reward system, and the frontal-limbic network. Decreased fALFF in the left ACC might be applied to differentiate the two subtypes of MDD.

## Data Availability Statement

The raw data supporting the conclusions of this article will be made available by the authors, without undue reservation.

## Ethics Statement

Ethical review and approval was not required for the study on human participants in accordance with the local legislation and institutional requirements. The patients/participants provided their written informed consent to participate in this study.

## Author Contributions

WG and GX designed the study. XC, YZ, and YO collected the original imaging data. FL, HL, JC, and JZ managed and analyzed the imaging data. YZ and XC wrote the first draft of the manuscript. All the authors contributed to and approved the final manuscript.

## Funding

This study was supported by grants from the National Natural Science Foundation of China (Grant Nos. 81771447 and 82171508), Natural Science Foundation of Hunan (Grant No. 2020JJ4784), Science and Technology Program of Hunan Province (Grant No. 2020SK53413), Key-Area Research and Development Program of Guangdong Province (2018B030334001), and Natural Science Foundation of Tianjin (Grant No. 18JCQNJC10900).

## Conflict of Interest

The authors declare that the research was conducted in the absence of any commercial or financial relationships that could be construed as a potential conflict of interest.

## Publisher's Note

All claims expressed in this article are solely those of the authors and do not necessarily represent those of their affiliated organizations, or those of the publisher, the editors and the reviewers. Any product that may be evaluated in this article, or claim that may be made by its manufacturer, is not guaranteed or endorsed by the publisher.
